# An Improved Character Recognition Framework for Containers Based on DETR Algorithm

**DOI:** 10.3390/s21134612

**Published:** 2021-07-05

**Authors:** Xiaofang Zhao, Peng Zhou, Ke Xu, Liyun Xiao

**Affiliations:** 1 Institute of Cognitive Computing and Intelligent Information, University of Science and Technology Beijing, Beijing 100083, China; 17863933393@163.com (X.Z.); xly15010521179@163.com (L.X.); 2 Research Institute of Artificial Intelligence, University of Science and Technology Beijing, Beijing 100083, China; 3 Collaborative Innovation Center of Steel Technology, University of Science and Technology Beijing, Beijing 100083, China; xuke@ustb.edu.cn

**Keywords:** character recognition, DETR (detection with transformers), split-attention, multi-scale location coding

## Abstract

An improved DETR (detection with transformers) object detection framework is proposed to realize accurate detection and recognition of characters on shipping containers. ResneSt is used as a backbone network with split attention to extract features of different dimensions by multi-channel weight convolution operation, thus increasing the overall feature acquisition ability of the backbone. In addition, multi-scale location encoding is introduced on the basis of the original sinusoidal position encoding model, improving the sensitivity of input position information for the transformer structure. Compared with the original DETR framework, our model has higher confidence regarding accurate detection, with detection accuracy being improved by 2.6%. In a test of character detection and recognition with a self-built dataset, the overall accuracy can reach 98.6%, which meets the requirements of logistics information identification acquisition.

## 1. Introduction

With the continuous development of international trade and the social economy, the port transportation industry has developed rapidly, and cargo throughput has increased dramatically. In order to ensure the real-time location of cargo and the safety of cargo during the logistics transportation process, containers are used as storage units to centrally detect, identify and track the information of the transported cargo. Therefore, the accurate detection and identification of container characters has begun to become an important part of the intelligent information management system for port logistics, and it is also an inevitable demand for the realization of comprehensive port automation. The traditional methods of port container character registration and identification are mainly based on manual identification, which is susceptible to large quantities of goods and bad weather and leads to low efficiency of number identification. With the object detection technology represented by convolutional neural networks constantly refreshing the highest detection index of each public image set, the application of machine vision in the industrial field has begun to expand [1,2].

There are three main categories of object detection methods based on convolutional neural networks: Faster R-CNN [3], YOLO series [4,5] and SSD [6]. Faster R-CNN performs best in terms of detection accuracy. On the one hand, it improves the accuracy of positioning and detection by means of two-stage detection. On the other hand, the existence of Region Proposal Network (RPN) makes positive and negative samples more balanced. However, these are also the two parts that bring a huge amount of calculation and time consumption to the detection process; therefore, it is unsuitable for industrial scenes that need high real-time requirements. The second method is the YOLO series, which is known for its rapid detection, and is a one-step detection model that maximizes the use of feature information. It has excellent real-time performance, but overall detection accuracy is not as good as Faster R-CNN. The last method is Single Shot MultiBox Detector (SSD), which combines the advantages of the above two methods. The model structure is simplified without loss of detection accuracy. However, this method is less effective for small target detection. The above three methods are all detection models obtained by innovations in model structure based on convolution operations. There are also many other similar models that are obtained by improving on these three models. However, the effect of model detection often depends on the characteristics of the training set samples, which is not ideal in situations where the scene changes in real time or the detected object changes in actual applications. For example, container characters abraded by long-term use, characters with distortion caused by an irregular angle of image acquisition and blurred images in inclement weather would make the characteristics of the target more diversified, and these characteristics cannot be fully learned in the training set. Changeable industrial scenes have higher requirements for the adaptability of model detection and feature depth mining. Therefore, the improvement of sample feature extraction capability is still a problem that object detection models must overcome.

For a deeper understanding of image information, researchers have tried to use transformers in the field of image processing, which have performed well in the field of natural language processing. Once the application of transformers in images was proposed, it quickly attracted the attention of scholars due to its excellent detection indicators in the public collection [7,8,9,10]. As a rising star in the field of vision, transformers quickly emerged in image classification [8], target detection and segmentation tasks [9] by virtue of their powerful feature extraction. The target detection task is represented by Facebook’s open-source DETR framework [10], which applied transformers to the target detection field for the first time. The basic idea is to treat the detection task as a problem of a prediction set and use the feature set to predict the set of bounding box, realizing true end-to-end detection under the premise of less prior information. The accuracy and operating efficiency of DETR detection results on COCO are basically the same as those of Faster R-CNN, and the effect is better than Faster R-CNN on large targets. The transboundary of transformers makes the visual field an effective detection tool besides CNN. Of course, no model is born versatile and excellent. Although the structure of DETR is simple and intuitive, because the transformer is set to obtain sufficient semantic information by increasing pixel attention, the extraction of target position information is limited to the sinusoidal encoding of the last layer of feature maps. Due to the single scale of location information, locating accuracy is not convincing enough in the actual application of the prediction frame detection process. Therefore, our work mainly revolves around the full acquisition of object location information on the basis of DETR.

Based on the above problems, the contributions of this paper are as follows:

1. The object detection algorithm DETR with transformers as the basic unit is introduced into the recognition of port container characters, which makes up for insufficient feature extraction and the poor anti-interference ability of model detection in the current widely used convolution operation.
2. In the backbone of the model, we introduce the split-attention structure on the basis of ResNet [11] and improve the feature extraction ability of the backbone through the connection method of multi-channel weight convolution.
3. With the introduction of multi-scale location coding (MSLC), the transformer structure no longer only focuses on the semantic information of input content, but gives consideration to both semantic and location information.

## 2. Materials and Methods

This paper improves on an end-to-end object detection framework, DETR. The original framework of DETR consists of three parts. The first part is feature extraction, including basic feature extraction and depth feature extraction, utilizing the two-dimensional feature map F from the ResNet network as the basic feature. The depth feature extraction is realized by splitting the summation of F and F’ results into a one-dimensional vector as the input of the encoder-decoder; among them, F’ is obtained by performing a sine-encoding operation on F. The second part outputs the prediction result, a position set and class set containing 100 prediction frames, through two FFN structures based on the extracted features, where 100 is manually set. The third part is to match the predicted result with the ground truth through the Hungarian algorithm. The boxes that do not contain the target and boxes with recognition error are matched with the background class, and the other boxes are matched with the ground truth.

**Figure 1:**
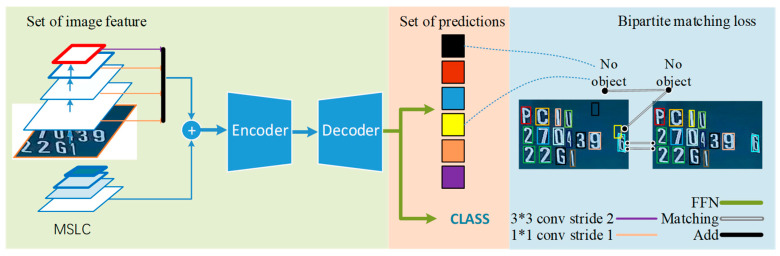
Container characters recognition framework based on the improved DETR framework.

We improve the detection model on the basis of DETR, and the improved detection framework is shown in Figure 1. Initially, in the basic feature extraction part, we introduce ResNeSt [12] instead of ResNet, which extracts image features in multiple scale spaces in the form of multi-channel split-attention. Secondly, in order to improve the accuracy of positioning and to obtain more location information, we output the result of the fusion of four feature maps of different scales in the backbone as the final feature F. Finally, in view of the problem that position coding of DETR loses some detailed information, we use multi-scale location coding to effectively improve the problem of large positioning deviation of the original DETR model.

The specific implementation steps of the model in this article are:

1. Obtain the multi-scale features of the image through the backbone named ResNeSt;
2. The sum of the multi-scale position code learned from the multi-scale feature map and the sine code of the feature map is used as the location information input of the transformer frame;
3. We pass each output embedding of the decoder to a shared feedforward network that predicts either a detection (class or bounding box) or a “no object” class, as shown in the pink color block in Figure 1;
4. Finally, the binary matching loss function based on the Hungarian algorithm is used to uniquely match the prediction result with the ground truth, thereby achieving parallel processing.

### 2.1. Backbone

As the basic feature extraction part of the model, the backbone is expected to fully mine the meaningful semantic information of the image. To this end, we use ResNeSt as our backbone [13,14,15].

Based on ResNet, our backbone uses a multi-channel feature map group containing split attention to replace the original bottleneck structure [13,14,15]. As shown in Table 1, the specific structural improvement includes three parts. The first part introduces the parameter *K*, that is, the input feature map is divided into *K* feature map groups according to channel dimension. The second part introduces a new hyperparameter *R* in each feature map group. This “*R*” specifies the number of slices of each feature map group. The computation result of all slices in each feature map group is obtained by fusing in the split-attention module. The split-attention module adopts the idea of SENet [13], and calculates the corresponding attention weight of each of the *R* slices by combining the attention of *R* slices after pooling, activation and other operations.

### 2.2. Multi-Scale Location Coding

We know that the self-attention module has the powerful ability to obtain local- and global-related information, but for object detection, the role of location information cannot be ignored. In the DETR framework, single-scale position information is imported into the model by adding the sine code of the position information of the feature points. Since deep features contain more semantic information, location information is not fully highlighted. Inspired by the scale-level encoding proposed in [16,17,18], we resize the feature map $p_{x}$ with different scales to the same size as the final feature map by max-pooling and sum it as the final multi-scale location coding. In the actual calculation, the sum of this part of the code and the sine code $PE_{Sine}$ [10] is used as the input of the position information of the transformer. The calculation process is:

(1) $$PE^{\prime }=PE_{Sine}+\overset{4}{\underset{x=1}{\sum }}maxPooling\left(p_{x}\right)$$

In addition, unlike the sine coding method that is calculated by formulas, the MSLC is randomly initialized and obtained by training and learning with the network.

### 2.3. Encoder-Decoder

The author in [19] once tested the natural language processing results of detectors with RNN, CNN and transformers as the basic operations to illustrate the comprehensive feature extraction capabilities of these three operations. Data form literature [19] are shown in Table 2. The experiment shows that the effect of the transformer is better than the other two, which shows the superiority of the transformer, whose superiority comes from the multi-head self-attention mechanism.

**Figure 2:**
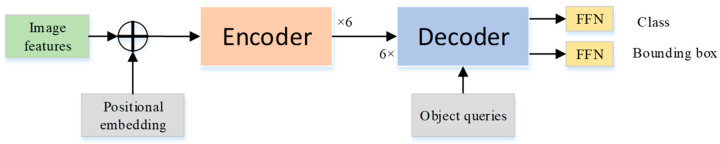
The structure of transformer.

The transformer framework used in this article consists of 6 encoders and 6 decoders. As shown in Figure 2, the encoder includes a multi-head self-attention mechanism and a fully connected feedforward network; both parts use residual connections. The structure of the decoder is similar to that of the encoder.

The difference is that, in addition to the output of the encoder, the input of the decoder also includes an object query vector used to indicate that the part of the object position information has been obtained in the feature map. This vector uses part of the learned information as prior knowledge and then passes through a labeled multi-head self-attention module. Subsequently, it is input to the decoder. The output of the decoder is the final feature extracted by the model, which is mapped to the set of class and the set of bounding box through two FFN modules.

### 2.4. Self-Attention

The attention mechanism obtains semantic information of input data by calculating similarity among pixels of input data. The multi-head attention method is used in the article to extract the semantic information of different dimensions such as colors, attributes and concepts. The self-attention calculation process is:

(2) $$Attention\left(Q,K,V\right)=softmax\left(\frac{QK^{T}}{\sqrt{d_{k}}}\right)V$$

**Figure 3:**
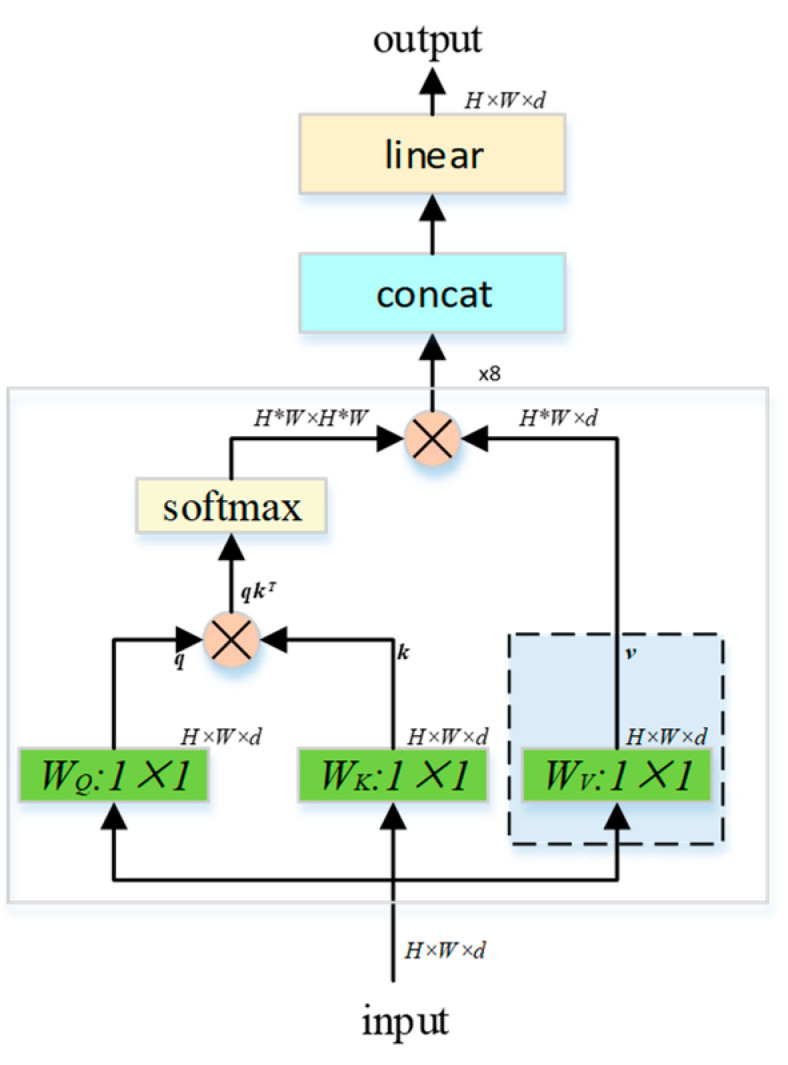
Multi-head attention.

From a mathematical point of view, *K*, *Q* and *V* are all linear transformations of input X, where *Q* represents query, *K* represents key, *V* represents value and *d_k_* is the dimension of *K*. The dot product of *Q* and *K* is to calculate the attention score between each pixel of the same input and different parts of the global, which represents the attention of a certain key point relative to the entire image. The normalized attention degree matrix is multiplied by *V* to obtain a weighting matrix, which is used to make the pixel pay attention to the part that it should pay attention to. The multi-head attention used in this paper adopts the parallel summation method of multiple self-attentions [20], and the specific calculation process is shown in Figure 3.

Here, $W_{Q}$, $W_{K}$ and $W_{V}$ are obtained according to the input through different linear transformations, where H and W are the width and height of the input, and d is the dimension of the input.

## 3. Experiments Setup

### 3.1. The Forward Process

In the last part of the detection framework, we use two feedforward networks composed of three-layer perceptron and linear calculation layers to predict bounding boxes and categories. Category information is obtained by predicting the output of FFN by the Softmax function. Position information is represented by the output of 100 fixed-size bounding boxes. Of course, this does not mean that there are 100 targets in the image to be detected. In the learning process, the final 100 prediction boxes are finally matched with the ground truth using the Hungarian algorithm [21], and the unmatched prediction boxes are automatically classified as the background category. This is why DETR does not need to use NMS (non-maximum suppression) in the output. The matching strategy is as follows:

(3) $$\hat{\sigma }=\underset{\sigma \in G_{N}}{\arg\min}\overset{n}{\underset{i}{\sum }}L_{Hungarian}\left(y,\hat{y}\right)$$

where $L_{Hungarian}(y,\hat{y})$ is the formula for calculating loss based on the matching result. When the predicted 100 targets are matched with real boxes one by one, the loss function can be used to optimize the system. The loss function includes cross-entropy classification loss and bounding box loss and is expressed as follows:

(4) $$L_{Hungarian}\left(y,\hat{y}\right)=\overset{N}{\underset{i=1}{\sum }}\left[-\log{\hat{p}}_{\hat{\sigma }\left(i\right)}\left(c_{i}\right)+{𝕝}_{\left\{c_{i}\neq \emptyset \right\}}L_{box}\left(b_{i},{\hat{b}}_{\sigma \left(i\right)}\right)\right]$$

where ${\hat{p}}_{\hat{\sigma }(i)}$ is the probability that the object is predicted to be class $c_{i}$, ${\hat{b}}_{\sigma \left(i\right)}$ represents the prediction box, $b_{i}$ represents the real box and $L_{box}\left(b_{i},{\hat{b}}_{\sigma \left(i\right)}\right)$ represents the bounding box regression loss, including GIoU Loss and L1 Loss, which calculates the center point and height difference between the prediction box and ground truth. The formula is as follows:

(5) $$L_{box}\left(b_{i},{\hat{b}}_{\sigma \left(i\right)}\right)=\lambda_{iou}L_{iou}\left(b_{i},{\hat{b}}_{\sigma \left(i\right)}\right)+\lambda_{L1}\|{\hat{b}}_{\sigma \left(i\right)}-b_{i}\|{}_{1}$$

Here,$L_{iou}(b_{i},{\hat{b}}_{\sigma (i)})$ is GIoU Loss [22], which represents the intersection over union (IoU) between prediction box and ground truth and is used to evaluate the accuracy of positioning.

### 3.2. Dataset

**Figure A1:**
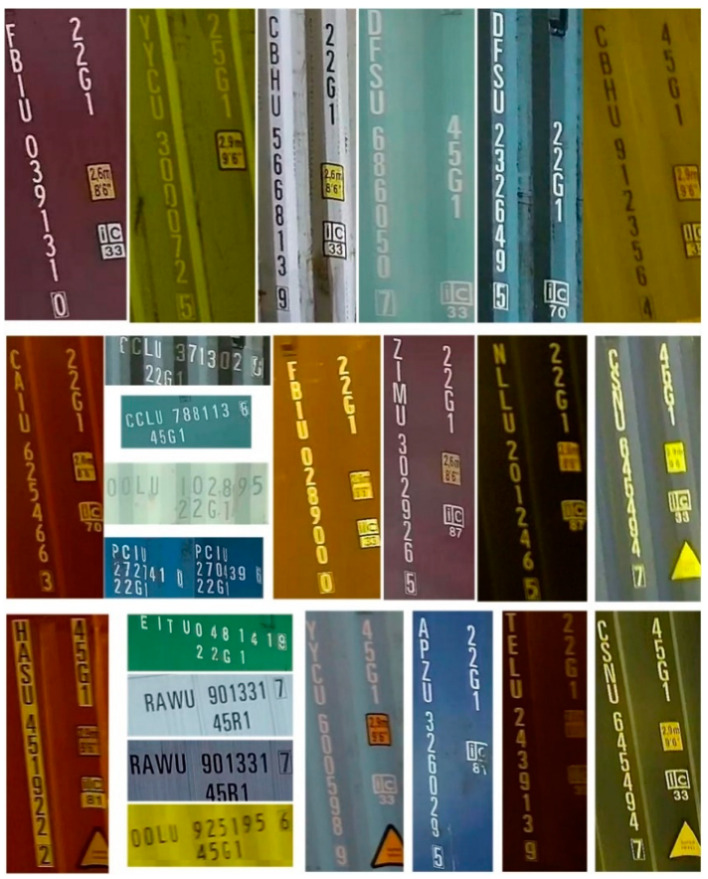
Some images of the self-built dataset in this paper.

The data set in this article is composed of container character images collected by the port. In order to avoid confusion of character detection by non-target numbers in the rest of the container, this part of the image has undergone region detection and cropping before detection. Part of the image of the data set is shown in Figure A1 of Appendix A. The container number is mainly composed of 11 characters; the first four digits represent the owner and operator of the container, the middle six digits represent the registration code of the container, and the last digit represents the check code. In addition to the 11-bit code, the target to be identified in this article also includes a 4-bit 95 code indicating the size of the box, which is used to confirm the box information.

Since the deep learning model has a strong dependence on data, considering that there will be rain, snow, fog, foreign objects and strong light exposure in actual application scenarios, we used the following methods to expand the data set:

(1) Random noise, filters and other methods were superimposed to simulate raindrop and snow movement trajectories on the image;
(2) Inspired by the literature [23], the following formula was used to simulate the effect of fog:
  (6) $$I\left(x,y\right)=A\rho \left(x,y\right)e^{-\beta d\left(x\right)}+A\left(1-e^{-\beta d\left(x\right)}\right)$$
  where $d\left(x\right)$ represents the distance between the pixel point and the center point of the image; β is the scattering coefficient of the atmosphere, which represents the scattering ability of the atmosphere in all directions per unit volume; $\rho \left(x,y\right)$ is the pixel point of the original image; and $I\left(x,y\right)$ is the output image of the simulated fog day;

(1) Performed random pixel block occlusion in the original image to simulate situations where characters are occluded by foreign objects;
(2) The brightness of the image was enhanced by the gamma transform algorithm and the adaptive white balance algorithm at the same time to simulate an environment illuminated by strong light.

**Figure 4:**
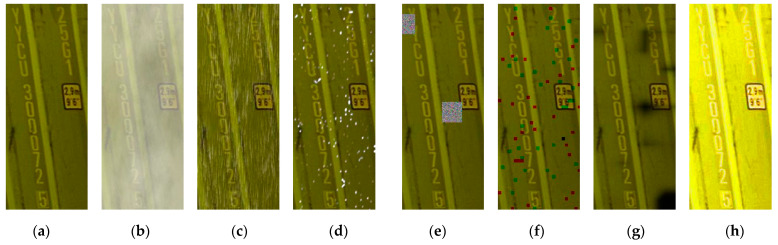
Data enhancement results of the sample set: (**a**) original drawing, (**b**) simulating foggy days, (**c**) simulating rainy days, (**d**) simulating snow days, (**e**) foreign bodies blocked 1, (**f**) foreign bodies blocked 2, (**g**) simulating smoke, (**h**) simulating strong light.

Some of the enhanced results are shown in Figure 4b–h. After data enhancement, the sample size reached 42,000, of which 33,000 were used as the training set, 4500 as the verification set and 4500 as the test set.

### 3.3. The Training Process

For the model mentioned in this paper, the number of training sets and the training environment are as follows:

**Training set:** 33,000;
**Training GPU:** NVIDIA Tesla V100 (deep learning framework version requirements, computing power ≥ 3.5);
**Memory:** 16 Gb × 1;
**Epoch:** 100.

**Figure 5:**
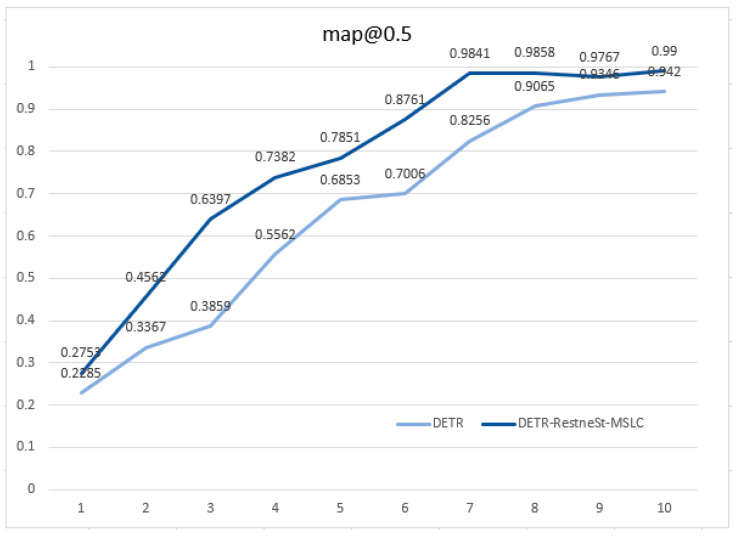
The mAP@0.5 of training 100 epochs.

During the training process, we recorded the mAP mean of every 10 epochs. The mAP curve of the DETR model and the model mentioned in this paper in the training process is shown in Figure 5. At the 70th epoch, the model tends to converge, and the mAP can reach 99%; the convergence speed and detection accuracy are both higher than in the DETR model.

## 4. Results Analysis

During the test, we evaluated the performance of the proposed DETR-ResNeSt-MSLC model and compared the performance with the original DETR model. It was found that for the detection results of the same object, our method made the model more willing to believe that it is the correct label.

**Figure 6:**
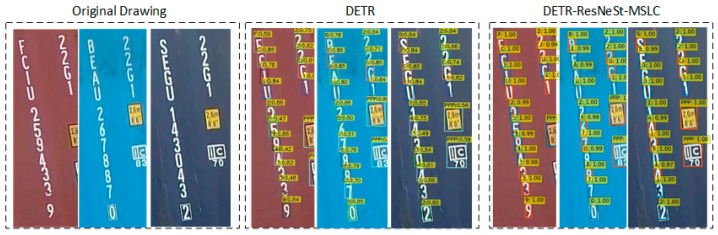
The detection results of the two models respectively on the characters.

This phenomenon can be verified by the higher confidence of object detection, as shown in Figure 6. The detection result confidence of DETR method is distributed between 0.4 and 0.9, and the detection confidence of DETR-ResNeSt-MSLC is greater than or equal to 0.98, which means that the improved model has the ability to better extract object features.

### 4.1. Test Results and Discussion Based on the Improved Model

Table 3 shows the comparison of the detection effect between the proposed model and DETR and DETR-ResNeSt without multi-scale position coding. We mainly selected the following evaluation indicators: mAP when IoU is 0.5, mAP of different IoU thresholds (from 0.5 to 0.95), recall and F1 Score.

The experimental results show that when the IoU was 50%, the detection effect of the ResNeSt backbone using multi-channel split-attention on the DETR model improved by 2.58% compared with the accuracy of DETR. When the IoU was between 50% and 95%, the DETR-ResNeSt model detection effect increased by 4.18%. The conclusion that the introduction of multi-channel split-attention in the backbone can effectively extract sample features and improve the accuracy of model detection is verified.

In order to prove the effectiveness of multi-scale location coding, we compared detection accuracy under different IoU requirements. When IoU = 0.5, the detection accuracy of the network including MSLC was 2.6% higher than that of the original sinusoidal location coding model, and when the average value of IoU was between 0.5 and 0.95, the accuracy was 2.8% higher. The recall rate of the model increased by 2.97%, and DETR-ResNeSt-MSLC was superior to the original model in recall rate and precision rate. This indicates that adding multi-scale location coding to the detection model can improve detection sensitivity of the model to position information, and the F1 score also indicates that the harmony average of error detection rate and over-detection rate is low.

The two improvements in the experiment have improved the feature extraction capability of the DETR-based object detection algorithm, expanded the research content of the transformer-based visual inspection field and also indicated that the transformer-based visual processing field will have unlimited possibilities in the future. In terms of application, this improved method provides new ideas for the detection of other similar application scenarios.

### 4.2. Discussion on the Experimental Results of Sample Reconstruction for Small Sample Classes

**Figure A2:**
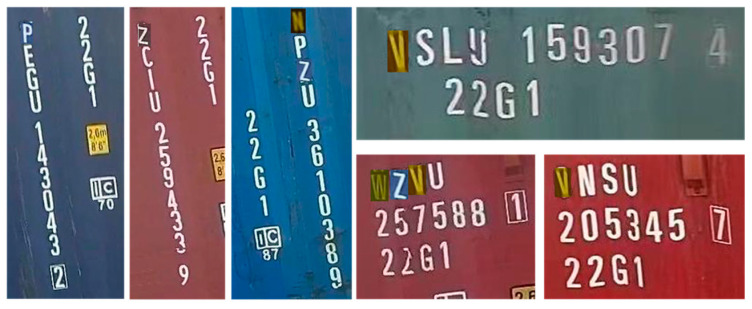
Some small sample character images after data reconstruction.

**Figure A3:**
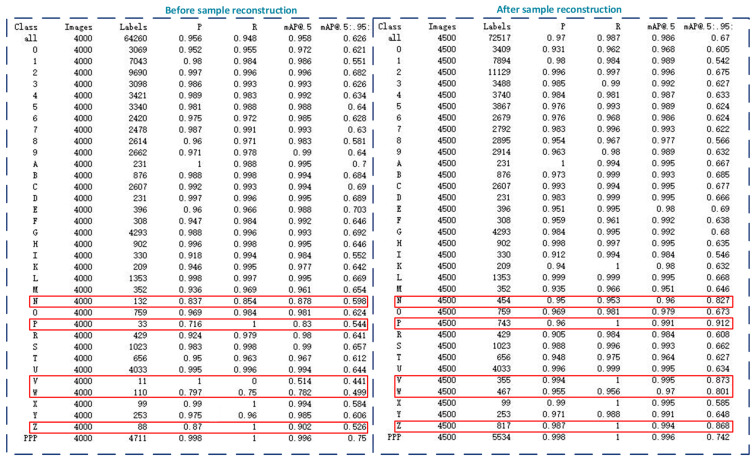
Comparison of category detection results after amplification of small sample characters.

In order to visually show the detection performance of each category, we analyzed the single-object detection results; relevant detection indicators are shown in Figure A2 in Appendix A. We found that although the data set had been enhanced by simulated weather samples, the number greatly improved; however, the problem of unbalanced samples in each category in the data set still had a greater impact on the overall map. For example, the number of samples “V” in the test set was only 11, which means that the original sample was only 1 before enhancement. This phenomenon is mainly caused by the imbalance of samples in each category. Based on the above problems, we performed data reconstruction for small sample categories, mainly by randomly overlaying the categories of mAP@0.5 < 0.95: Z, W, V, P, N on the background of other samples to obtain reconstructed images. The image is shown in Figure A3 in Appendix A. In this way, we expanded the data set by randomly covering more than one to five categories in the background of some samples and obtained 500 newly reconstructed samples. The sample increment of each category and the test results after small sample enhancement are shown in Table 4.

It can be seen that after sample reconstruction, the number of small sample categories increased, and the detection accuracy was improved, which contributed 2.8% to the overall detection accuracy.

Data are the basis for deep learning methods to work effectively. Data enhancement in practical applications needs to consider the impact of scene changes on the data and the balance of the data volume at the same time. The filtering-based data enhancement method used in this paper can effectively solve the problem of insufficient data sample size under severe outdoor weather. The small sample class reconstruction method proposed in this paper can deal with the problem of imbalance in the number of samples in the multi-class identification problem. This method also provides ideas for applications in related fields.

## 5. Conclusions

This paper proposes an end-to-end recognition framework for container characters based on the DETR-ResNeSt50-MSLC model. The test result in the self-built container character data set can reach 98.6%, which meets the requirements of the smart port for the detection accuracy of container numbers. It can be used for logistics and transportation monitoring and can avoid accidental losses to a large extent. On the one hand, this paper introduces the backbone with the multi-channel split-attention module into the transformer-based detection model to achieve full extraction of character features, and the detection result is 2.58% higher than the original DETR model. On the other hand, multi-scale location coding is introduced to improve the model’s ability to detect and locate objects.

However, the model mentioned in this article still has the problem of large calculations due to the global mining of image features by the transformer, which makes our model converge slowly, as shown in Figure 5 in Section 3. In fact, for an image, we often only pay attention to the features of the part where the objects exists, and we do not need to perform feature mining on the entire image. Therefore, how to focus on effective feature extraction and accelerated model convergence will be the direction of our follow-up research.

## Figures and Tables

**Table 1:** The structure composition of the modified backbone and the original DETR backbone.

**Layer Name**	Output Size	ResNet-50	ResNeSt-50
Conv1_x	112×112	7×7.64. stride 2	
Conv2_x	56×56	3×3 max pool. stride 2	
		$\left\{\begin{array}{c} 1\times 1.64\\ 3\times 3.64\\ 1\times 1.256 \end{array}\right\}\times 3$	concat1×1.64/KR3×3.64/K×RSplit Attention…1×1.64/KR3×3.64/K×RSplit Attention⏟K 1×1.256×3
Conv3_x	28×28	$\left\{\begin{array}{c} 1\times 1.128\\ 3\times 3.128\\ 1\times 1.512 \end{array}\right\}\times 3$	concat1×1.128/KR3×3.128/K×RSplit Attention…1×1.128/KR3×3.128/K×RSplit Attention⏟K 1×1.512×3
Conv4_x	14×14	$\left\{\begin{array}{c} 1\times 1.256\\ 3\times 3.256\\ 1\times 1.1024 \end{array}\right\}\times 3$	concat1×1.256/KR3×3.256/K×RSplit Attention…1×1.256/KR3×3.256/K×RSplit Attention⏟K 1×1.1024×3
Conv5_x	7×7	$\left\{\begin{array}{c} 1\times 1.512\\ 3\times 3.512\\ 1\times 1.2048 \end{array}\right\}\times 3$	concat1×1.512/KR3×3.512/K×RSplit Attention…1×1.512/KR3×3.512/K×RSplit Attention⏟K 1×1.2048×3
	1×1	Average pool. 1000-d fc. softmax	

**Table 2:** Comprehensive feature extraction ability comparison of RNN/CNN/transformer [19]. 2014 and 2017 are the test set of NLP which can be found in the literature [19]; DE-EN: German to English; Acc means accuracy on the test set.

Model	DE->EN		
	2014	2017	Acc (%)
RNNS2S	29.1	30.1	84.0
ConvS2S	29.1	30.4	82.3
Transformer	32.7	33.7	90.3

**Table 3:** Detection results of different models in the test set.

Model	mAP@0.5 (%)	mAP@0.5:0.95 (%)	Recall (%)	F1 Score (%)
DETR	93.42	60.02	95.6	93
DETR-ResNeSt	96	64.2	96.03	94
DETR-ResNeSt-MSLC	98.6	67	98.7	98

**Table 4:** Sample category and detection accuracy before and after small sample class enhancement.

Class		V	P	N	Z	W	ALL
Before	Quantity	11	33	132	88	110	4000
	mAP@0.5	0.514	0.83	0.878	0.902	0.782	0.958
After	Quantity	355	743	454	817	467	4500
	mAP@0.5	0.995	0.991	0.96	0.994	0.97	0.986

## Data Availability

Data sharing is not applicable to this article.

## Appendix A

Explanatory materials and some experimental results of the data set are presented in this paper.
